# Cellular and molecular insights into incomplete immune recovery in HIV/AIDS patients

**DOI:** 10.3389/fimmu.2023.1152951

**Published:** 2023-05-02

**Authors:** Liting Yan, Kaiju Xu, Qing Xiao, Lin Tuo, Tingting Luo, Shuqiang Wang, Renguo Yang, Fujie Zhang, Xingxiang Yang

**Affiliations:** ^1^Department of Infectious Disease, Sichuan Provincial People’s Hospital, University of Electronic Science and Technology of China, Chengdu, China; ^2^Chinese Academy of Sciences Sichuan Translational Medicine Research Hospital, Chengdu, China; ^3^Clinical and Research Center for Infectious Diseases, Beijing Ditan Hospital, Beijing, China

**Keywords:** human immunodeficiency virus, incomplete immune recovery, immunological nonresponders, immunocytes, soluble molecules

## Abstract

Highly active antiretroviral therapy (ART) can effectively inhibit virus replication and restore immune function in most people living with human immunodeficiency virus (HIV). However, an important proportion of patients fail to achieve a satisfactory increase in CD4^+^ T cell counts. This state is called incomplete immune reconstitution or immunological nonresponse (INR). Patients with INR have an increased risk of clinical progression and higher rates of mortality. Despite widespread attention to INR, the precise mechanisms remain unclear. In this review, we will discuss the alterations in the quantity and quality of CD4^+^ T as well as multiple immunocytes, changes in soluble molecules and cytokines, and their relationship with INR, aimed to provide cellular and molecular insights into incomplete immune reconstitution.

## 1 Introduction

Highly active antiretroviral therapy (ART) significantly reduces human immunodeficiency virus (HIV) or acquired immune deficiency syndrome (AIDS) related morbidity and mortality (1). After ART initiation, the plasma viral load drops to an undetectable level and the immune function gradually recovers to an approximately normal level in most individuals (2). However, an important proportion of HIV/AIDS patients, about 8-42%, persistently maintain low CD4^+^ T cell counts despite continuous virological suppression after at least two years of ART (3). These patients are referred to as immunological non-responders (INRs) (4), and this state is called incomplete immune reconstitution, or immunological nonresponse (INR) (4, 5).

Currently, there is no consensus on the definition of INR. According to previous reports, CD4^+^ T cell counts and ART time were the best features to describe INR, and the most frequent criterion was CD4^+^ T cell count <350 cells/µL after ≥24 months of virological suppression (5, 6). Some researchers also defined INR with CD4^+^ T cell count less than 200, 250, 400, or 500 cells/µL (6). Persistent low CD4^+^ T cell levels in these patients lead to an increased incidence of AIDS and non-AIDS events, such as metabolic syndrome, cardiovascular disease, liver disease, neurocognitive impairment, and malignant tumors, which in turn increase the risk of mortality (7, 8).

The occurrence of INR in HIV/AIDS patients may be affected by multiple factors, mainly including decreased bone marrow hematopoiesis, insufficient thymus output, residual virus replication, co-infection during ART, intestinal flora translocation, abnormal immune activation, type of antiretroviral regimen, baseline CD4^+^ T cell levels, age, sex, and genetic characteristics (4, 9). However, the precise mechanisms underlying INR remain an extremely challenging issue. Several characteristics of immunocytes may provide insights into these mechanisms. Therefore, we conducted this review to outline the cellular and molecular alterations associated with INR from an immunological perspective.

## 2 Quantity and quality of T cells

### 2.1 Overview of T cells

**Figure 1:**
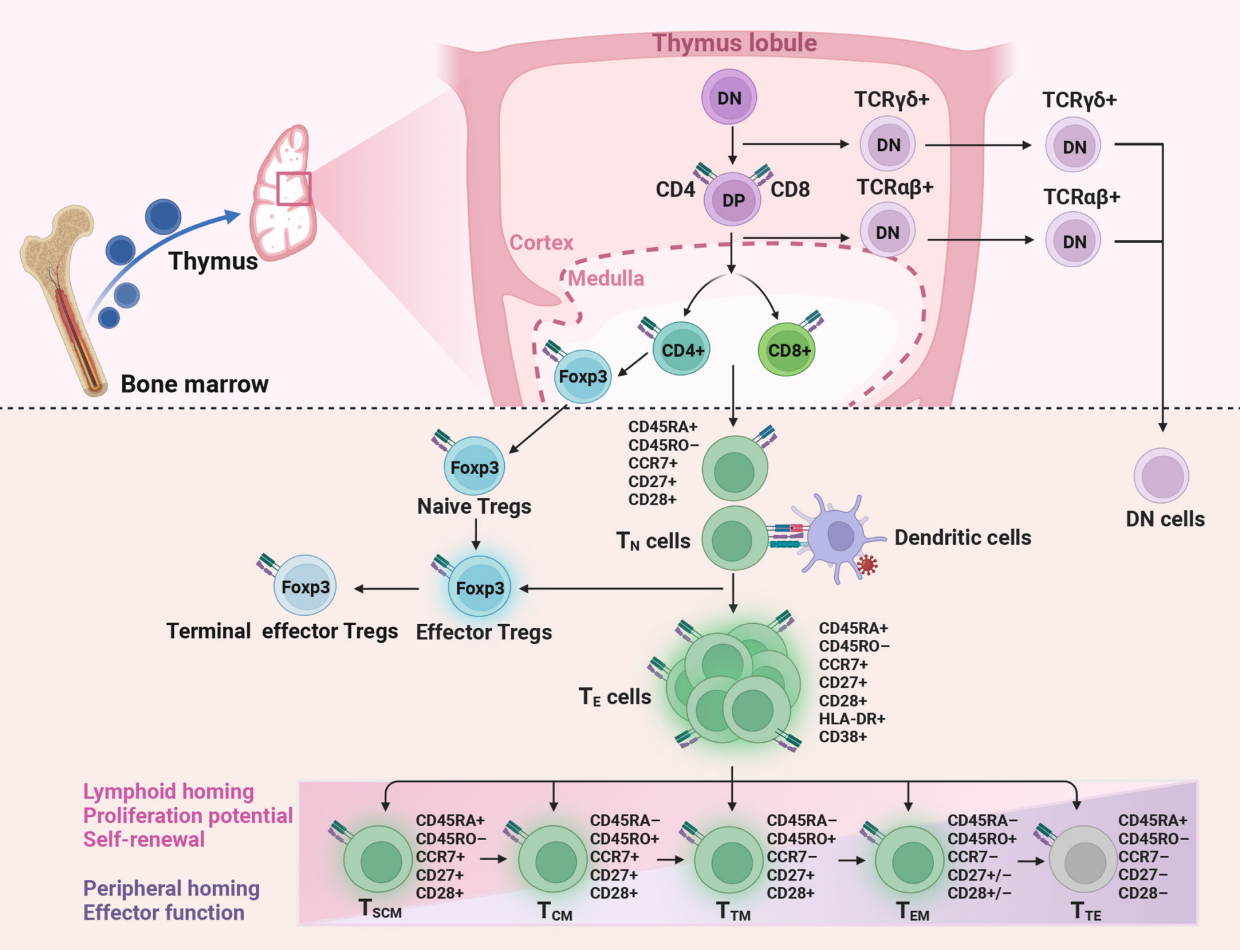
Production and differentiation of T cells. Naive CD4^+^ and CD8^+^ T (T_N_), naive regulatory T (Tregs) cells, and double-negative (DN) T cells are released from the thymus. Then CD4^+^ and CD8^+^ T cells get activated and differentiate into effector (T_E_) cells after encountering antigens. Only a small part of T_E_ develops into memory T cells, including stem-like memory (T_SCM_), central memory (T_CM_), transitional memory (T_TM_), effector memory (T_EM_), and terminal effector (T_TE_) cells. In HIV immunological non-responders, the number of T_N_ cells produced by the thymus is reduced, while the proportion of activated T cells is increased. DP, double-positive; TCRαβ, T cell receptor alpha and beta; TCRγδ, T cell receptor gamma and delta; Foxp3, factor forkhead box P3.

T cells are the major components of the adaptive immune system. They are released from the thymus as mature naive T (T_N_, CD45RA^+^CCR7^+^CD27^+^CD28^+^) cells with a given epitope specificity after positive and negative selection (**Figure 1**) (10, 11). During pathogen infection or vaccination, T_N_ cells get activated and differentiate into effector cells (T_E_), accompanied by the acquisition of effector function, altered tissue homing, and robust proliferation function to expand in number (12–14). Following antigen clearance, T_E_ cells undergo a contraction phase and only a small portion develop into long-lived memory T cells (12). The gene expression, phenotypic and functional properties of memory T cell subsets suggest that these cells differentiation follows a linear progression along a continuum of major clusters, including stem-like memory (T_SCM_, CD45RA^+^CD45RO^–^CCR7^+^CD27^+^CD28^+^) cells, central memory (T_CM_, CD45RO^+^CCR7^+^CD27^+^CD28^+^) cells, transitional memory (T_TM_, CD45RO^+^CCR7^–^CD27^+^CD28^+^) cells, effector memory (T_EM_, CD45RO^+^CCR7^–^, CD27^–^CD28^+^ for CD4^+^ T and CD27^+^CD28^–^ for CD8^+^ T) cells, and terminal effector (T_TE_, CD45RA^+^CCR7^–^CD27^–^CD28^–^) cells (10). Among them, the cells with lower differentiation degrees give rise to more differentiated progeny in response to antigen stimulation or potential homeostatic signaling (10, 15, 16). For instance, T_SCM_ cells are able to self-renew and generate all memory subsets (15, 16), T_CM_ cells have the potential to home to secondary lymphoid tissue and are capable of generating T_EM_ cells *in vitro* (17), T_TE_ cells are terminally differentiated cells with low proliferative and functional capacity (18, 19).

The life journey of a T cell includes quiescence, ignorance, anergy, exhaustion, senescence, and death, which is regulated by tolerance checkpoints to protect the body from hyperinflammation and autoimmunity (20). At the T_N_ cells stage, quiescence and ignorance act as checkpoints to actively maintain tolerance (21, 22). At the effector stage, exhaustion and senescence can limit excessive inflammation and prevent immunopathology (20). However, during chronic infection or cancer, persistent antigenic stimulation or inflammation causes T cells to enter a progressive exhaustion state (23). Exhausted T cells are characterized by increasing loss in effector function coinciding with increased expression of immune checkpoint inhibitors, decreased secretion of cytokines, altered transcriptional as well as epigenetic programs, and poor memory recall as well as homeostatic self-renew (20).

### 2.2 CD4^+^ T cells

#### 2.2.1 Decreased production

In HIV-INRs, the number of CD4^+^ T_N_ cells and mature CD4^+^ T cell subsets were repeatedly observed to be significantly reduced (24–27). In general, the number of T cells is regulated by a dynamic balance between the production, destruction, and trafficking of lymphocytes between peripheral circulation and lymphoid organs (28). The thymus is crucial for the generation of T cells, and thymic function is usually assessed by T cell receptor excision circles (TRECs), recent thymus emigrants (RTEs), or T_N_ cell counts. Previous studies have shown that the RTEs CD4^+^ T cells and the signal joint (sj)/β TREC ratios can predict disease progression in HIV-infected patients (29, 30). Moreover, the increase of T_N_ cells, the proportion of RTEs in CD4^+^ T cells, the number of sj-TRECs, and the sj/β TREC ratios were significantly lower in INRs than in immunological responders (IRs) (31, 32). Our recent study also found that the CD4^+^ T_N_ cells, RTEs CD4^+^ T cells, and TRECs were remarkably lower in INRs compared to complete responders and healthy subjects (33). These findings suggest that the thymus output of T_N_ cells is significantly reduced in INRs, which play an important role in incomplete immune reconstitution.

In addition, HIV-infected individuals often have impaired mitochondrial function, resulting in diminished cellular metabolic activity and asymmetric mitochondrial distribution during cell division in which cells that received old mitochondria would be short-lived compared to cells that had newly synthesized mitochondria (34, 35). It is likely that INRs are incapable of generating memory long lived CD4^+^ T cells due to the asymmetric distribution of mitochondria in dividing cells (34). Moreover, mitochondrial dysfunction may also affect the regenerative potential of memory CD4^+^ T cells (36). And the low-productive proliferation of memory CD4^+^ T cells is linked to impaired immune restoration (36).

#### 2.2.2 Increased destruction

In terms of T cells destruction, previous researchers found that a low nadir CD4^+^ T cell count was associated with high CD4^+^ T cell apoptosis (37), and the expression of apoptotic markers such as caspase-3, annexin-V, and proapoptotic proteins in INRs was significantly higher than that in IRs (38), indicating that apoptosis plays a major role in CD4^+^ T cell depletion (38, 39). In recent years, INRs and IRs were reported to have no statistical differences for RTEs CD4^+^ T cells in early and late apoptosis (40). However, INRs had a higher dead RTEs CD4^+^ T cells percentage driven by pyroptosis than IRs, and RTEs CD4^+^ T cells death by pyroptosis was significantly higher than by apoptosis in INRs (40). Pyroptosis is regulated by inflammasome-mediated caspase-1 activation and the release of interleukin-1β (IL-1β) and IL-18. The nucleotide-binding oligomerization domain (NOD)-like receptor (NLR) family (41), pyrin domain containing 3 (NLRP3) inflammasome is one of the prevalently studied among inflammasomes (41). According to a recent study, NLRP3 and IL-18 genes were significantly upregulated in INRs compared to IRs (42). Another study found that high-level expression of Caspase-1 and IL-18 were associated factors that affect the reconstruction of immune function (43). However, these two studies only showed the association of NLRP3, Caspase-1, and IL-18 with incomplete immune reconstitution. Zhang et al. identified that the NLRP3 inflammasome drives caspase-1 activation and pyroptosis in CD4^+^ T cells through a mechanism dependent on ROS production, suggesting that NLRP3-dependent pyroptosis plays an essential role in CD4^+^ T cell loss in chronically HIV-infected patients (44). Moreover, our recent study found that CD4^+^ T are prone to ferroptosis, which may be a novel way of increasing CD4^+^ T cell destruction (33). Collectively, these studies indicate the increase of CD4^+^ T cell destruction mediated by apoptosis, pyroptosis and ferroptosis may be an important cause of incomplete immune reconstitution.

#### 2.2.3 Increased CD4^+^ T cell activation

Although HIV-INRs are characterized by significant decreases in the total number of CD4^+^ T cell counts (6, 45), the frequency of cycling CD4^+^ T cells is increased, and CD4^+^ T cells are more activated (**Table 1**) (25, 27, 56, 57). Using CD71 as a marker for cycling T cells, the researchers found that the significantly increased cycling cells in INRs included both CD4^+^CD45RA^+^ and CD4^+^CD45RA^–^ subsets compared to IRs and healthy subjects (27), whereas IRs showed an increased frequency of CD4^+^CD71^+^CD45RA^–^ subset with a significant decrease in CD4^+^CD71^+^CD45RA^+^ subset compared to healthy subjects (27). Lederman et al. found that proportions of cycling CD4^+^ T_N_ cells were comparable among INRs, IRs, and healthy controls, proportions of cycling CD4^+^ T_CM_ and T_EM_ cells were significantly greater in INRs than in IRs and in healthy controls (25). In addition, cycling CD4^+^ T cells from healthy subjects and IRs can complete cell division *in vitro*, while cycling CD4^+^ T cells from INRs have mitochondrial dysfunction and are unable to complete cell division (58), suggesting that the function of cycling CD4^+^ T cells is impaired in INRs. Using CD38 and human leukocyte antigen (HLA)-DR to reflect T cell activation, Massanella et al. found that INRs exhibited a significantly increased frequency of HLA-DR expressing cells compared to IRs (46). Notably, INRs showed a significantly lower percentage of CD38^+^CD45RA^+^CD4^+^ T cells and a significantly higher percentage of CD38^+^CD45RA^–^CD4^+^ T cells (46), indicating a high level of activation in CD45RA^–^CD4^+^ T cells. In greater detail, the frequency of each subset in the CD45RA^–^CD4^+^ T cell compartment showed a significant increase in T_TM_, T_EM_, and T_TE_ cells in INRs (47), while T_CM_ cells showed similar levels in INRs, IRs, and healthy subjects (47). Except for the alterations in memory CD4^+^ T cells differentiation for INRs, their function may also be impaired. It’s reported that the metabolic activity of activated memory CD4^+^ T cells derived from INRs was reduced, which may lead to low regenerative potential (36). And the transition from T_CM_ to T_TM_ cells was found to be not completely normalized, and T_CM_ cell death increased *in vitro* in INRs (47).

**Table 1:** Alterations in the quantity of immunocytes in HIV/AIDS patients with incomplete immune reconstitution.

Immunocyte	Total	T_N_	T_E_	T_CM_	T_EM_	T_TE_	Reference
CD4^+^ T cells	↓	↓	↓	↓	↓	↓	(24–27)
Cycling CD4^+^ T cells	↑	NS	↑	↑	↑	NM	(25, 27)
Activated CD4^+^ T cells	↑	↓	NM	NS	↑	↑	(46, 47)
CD8^+^ T cells	↑	↓	NM	↑	↑	↑	(25, 26, 48)
Activated CD8^+^ T cells	↑	NM	NM	NM	NM	NM	(25, 40)
Frequency of Tregs	↑	↑	↑	NA	NA	NA	(49–53)
Absolute number of Tregs	↓	NM	NM	NA	NA	NA	(51, 52)
DN T cells	↓	NA	NA	NA	NA	NA	(54, 55)

NS, not significant; NM, not mentioned; NA, not applicable.

↓, decreased; ↑, increased.

#### 2.2.4 Mechanisms of immune activation

The increased CD4^+^ T cell cycling and activation observed in INRs reflects extensive immune activation. It is firmly established that despite the effective suppression of HIV replication by ART, people living with HIV still present persistent chronic immune activation and systemic inflammation (59–61). This condition is driven by various factors, including persistent HIV viral reservoir (28), low residual viremia (62), co-infections (63, 64), microbial translocation and dysbiosis (65–67). As expected, the HIV reservoir was higher in INRs than IRs (68), and high levels of cell-associated RNA and proviral DNA were associated with lower CD4 counts (68, 69). Similar results were found by Scherpenisse et al. that the cell-associated HIV un-spliced RNA to multiply-spliced RNA ratio at 12 weeks of ART was negatively predicted CD4^+^ T cell counts at 48 and 96 weeks (70). However, the association between residual viremia and immune reconstitution is controversial. Some researchers found that very low-level viremia was not associated with INR (49, 71), and attempts to target residual viral replication in INRs have not yielded a decisive benefit in restoring CD4^+^ T cell counts (72, 73). Another study found that viral blips had no significant impact on immune reconstitution, whereas persistent detectable viremia and virological rebound to ≥5000 copies/mL were associated with arrested immune reconstitution (74). These findings suggest that the impact of low-level viremia on CD4^+^ T cell restoration may be related to the residual viral load levels. For intestinal microecology, plasma levels of bacterial ribosomal 16S RNA, an index of microbial translocation from the gastrointestinal tract, are correlated with the magnitude of immune restoration in HIV-infected patients on ART (75). Moreover, HIV infection alters the composition of intestinal flora and reduces its diversity, which is not normalized after the introduction of ART (76), and is closely associated with immune dysfunction (77). For example, a higher abundance of Fusobacterium (78), a reduced abundance of Ruminococcaceae (79), the relative abundances of unclassified Subdoligranulum sp. and Coprococcus comes (80), were demonstrated to be associated with poorer CD4^+^ T cell recovery following ART. To sum up, these studies indicate that hard-to-eliminate viral reservoirs, persistent detectable viremia, microbial translocation, intestinal flora imbalance, and co-infections contribute to excessive activation of T cells, which impair immune reconstitution. It is worth noting that the above factors are not the direct cause of incomplete immune reconstitution, and there may be individual differences in the primary causes of T cell overactivation.

#### 2.2.5 Increased CD4^+^ T cell exhaustion

Persistent chronic immune activation and systemic inflammation contribute to the development of T cell exhaustion (81). In HIV infection, the expression of programmed death-1 (PD-1) on virus-specific T cells is a major marker of exhaustion (82). Other inhibitory receptors include T cell immunoglobulin and ITIM domain (TIGIT), cytotoxic T lymphocyte antigen-4 (CTLA-4), lymphocyte activation gene protein (LAG3), T cell immunoglobulin domain and mucin domain-containing protein 3 (TIM3), 2B4 (CD244) and CD160 (81). Among them, PD-1, TIGIT, LAG3, and TIM3 are common regulators of exhaustion on HIV-specific CD4^+^ and CD8^+^ T cells (83–86), CTLA-4 is more selectively upregulated on exhausted CD4^+^ T cells (87), and 2B4 as well as CD160 are characteristically upregulated on exhausted CD8^+^ T cells while low expression on exhausted CD4^+^ T cells (88). For CD4^+^ T cell exhaustion-related markers, researchers found that they were tightly correlated with the size of the T cell viral reservoir (89, 90), the decrease of CD4^+^ T cell counts, and disease progression (82, 87, 91). For example, in HIV elite controllers who can spontaneously control viral replication in the absence of ART and maintain a high CD4^+^ T cell count, the co-expression pattern of PD-1, TIGIT, and CTLA-4 was similar to healthy controls, and significantly lower than those of subjects receiving ART (85). Furthermore, the percentage of co-expression of inhibitory molecules on memory CD4^+^ T cells significantly negatively correlates to CD4 count and CD4/CD8 ratio (85). Cockerham LR et al. also found that PD-1 expression on CD4^+^ T cells was associated with CD4^+^ T cell activation and inversely with CD4^+^ T cell counts in patients on ART (69, 92). Similarly, patients with incomplete immune reconstitution despite successful ART express significantly higher levels of PD-1 than patients with normal recovery of CD4^+^ T cells (27, 93, 94). These studies indicate that inhibitory receptors mediated T cell suppression may be involved in the development of impaired immune reconstitution in HIV patients. However, the specific regulatory mechanism needs to be further studied.

Exhausted CD4^+^ T cells exhibit reduced proliferative capacity and helper functions, and a decreased production of cytokines such as IL-2 and interferon-gamma (IFN-γ) (81, 95, 96). Moreover, cell apoptosis is positively correlated with the level of PD-1 expression, indicating that exhausted CD4^+^ T cells from INRs are prone to apoptosis (27).

#### 2.2.6 Increased CD4^+^ T cell senescence

Aside from T cell exhaustion, chronic viral infection and inflammation further induce immune senescence (27, 97, 98). The senescent T cell phenotype is marked by a lack of CD28 expression, a decrease in homing receptors (such as CD62L and CCR7), and an increase in the expression of the senescence marker, CD57 (99). In HIV-infected patients on ART, there was a significant negative correlation between the absolute count of the CD4^+^ T_N_ cell subset and the expression of CD57 (26). Also, HIV-INRs displayed an increased frequency of CD57^+^ cells in total CD4^+^, CD4^+^CD45RA^+^, and CD4^+^CD45RA^–^ cell subsets, and cycling as well as non-cycling CD4^+^ T cells compared to IRs (27). These results indicate that CD4^+^ T cells from INRs are generally more activated, exhausted, and senescent despite successful control of viral replication (26, 27). However, senescent cells exhibit telomere loss, mitochondrial compromise, cell cycle arrest, activation of pro-inflammatory secretory pathways, and limited proliferation in response to antigen stimulation (27, 99, 100). And the higher the expression of CD57 in CD4^+^ T cells, the lower their proliferative capacity (27).

### 2.3 Regulatory T cells

Regulatory T cells (Tregs) expressing the transcription factor forkhead box P3 (Foxp3) are naturally produced in the thymus as a functionally mature subpopulation of CD4^+^ T cells (nTregs) and can also be induced from T_N_ cells (iTregs) after encountering of antigens in the periphery (101, 102). They can suppress the proliferation of T_N_ cells, the differentiation from T_N_ to T_E_ cells, the effector activities of differentiated T cells, and can also suppress the functions of B cells, natural killer (NK) cells, NKT cells, as well as antigen-presenting cells (103–105). Thus, Tregs are indispensable for the maintenance of self-tolerance and immune homeostasis (103).

The role of Tregs in the pathogenesis of HIV infection has been extensively debated. They can play a beneficial role by inhibiting T cell activation and HIV replication in CD4^+^ T cells (106, 107), or play a harmful role by inhibiting HIV-specific CD4^+^ and CD8^+^ T cell responses (108, 109), aggravating lymphatic tissue fibrosis (110), and contributing for intestinal flora translocation (111). More importantly, dysregulation of homeostasis in Tregs may hamper immune reconstitution. According to several studies, HIV-INRs have a significantly higher percentage of Tregs within the CD4^+^ T cells (49–53), including total, naïve, effector, and terminal effector Tregs (49, 50), together with a drop in the absolute number of Tregs and a decrease in HIV-specific immunosuppressive functions (51, 52). A higher percentage of Tregs is associated with a reduced thymic output of CD4^+^ T_N_ cells (52). Méndez-Lagares et al. also found a negative correlation between Tregs and CD4^+^ T_N_ cells (51). Moreover, a recent study proposed that the failure of INRs to restore CD4^+^ T cells as a consequence of defective Treg survival and function, resulting in a phenotype of uncontrolled cycling, immune exhaustion, and increased cell death (58). As for the effect of baseline Tregs, one study found that Tregs percentage at baseline was a strong independent prognostic factor of immune recovery, and a 1% increase of initial Tregs percentage was associated with a 1.9% lower CD4^+^ T cell counts at month 24 (112). In contrast, another study found no effect of Tregs percentages at baseline was detected on CD4^+^ T cells recovery (113). These studies suggest that although the impact of baseline Tregs on immune reconstitution is controversial, a high percentage and functional defects of Tregs during antiretroviral therapy have a negative impact on immune reconstitution. Furthermore, it is necessary to validate the potential value of Tregs in HIV-INRs in larger sample cohorts because of the limited number of current studies and sample sizes.

### 2.4 CD8^+^ T cells

CD8^+^ T cells are a critical component of the cellular immune response to viral infections. In HIV-infected patients, CD8^+^ T cells play an important role in the control of HIV replication and the HIV reservoir (114–116). Additionally, for patients with CD4^+^ T cells above 500 cells/μL after long-term ART, high CD8^+^ T cell counts are associated with CD4 recovery (117). However, in incomplete immune reconstitution, the quantity and quality of CD8^+^ T cells are disordered. According to previous studies, the absolute numbers of CD8^+^ T_N_ cells were significantly lower in INRs than that in IRs and healthy subjects, while circulating T_CM_, T_EM_, and T_TE_ cells were higher in INRs (25). Méndez-Lagares et al. also found that the frequency of the CD8^+^ T_N_ cell subset was significantly lower in INRs, and the CD8^+^ T_EM_ cells showed a significant expansion in INRs when compared with the IRs (26). In fact, the total number of CD8^+^ T cells is consistently elevated even after long-term ART in HIV-infected patients (48). Additionally, when the low CD4^+^ T cells patients were included, the high CD8^+^ T counts were associated with a poor increase in CD4^+^ T cells during ART (48). These results can be attributed to the absence of CD4^+^ T cells help on the one hand (117), and the activation and exhaustion of CD8^+^ T cells on the other hand (118). As the study reported, CD8^+^ T cell counts were positively correlated with the viral reservoir (119–121), and the elevation of CD8^+^ T cells is associated with immune activation and increased immune anergy (48, 122). Elite controllers possessed a significantly lower level of activated HIV-specific CD8^+^ T cells than non-controllers (123), while INRs possessed a higher level of activated CD8^+^ T cells (CD38^+^, HLA-DR^+^) than IRs (25, 40). Hunt et al. found that for every 5% increase in the proportion of activated CD8^+^ T cells mean 35 fewer CD4^+^ T cells were gained (124). These studies indicate that the extensive expansion, activation, and exhaustion of CD8^+^ T cells further contribute to CD8 accumulation over disease progression, which plays a role in incomplete immune reconstitution.

The exhaustion of CD8^+^ T cells is a progressive condition that starts with an initial loss of proliferation, cytotoxic potential as well as decreased IL-2 production, and eventually loss of the ability to produce IFN-γ in more pronounced stages (83). Therefore, exhausted CD8^+^ T cells in HIV-infected patients lose their capacity to kill efficiently infected cells (83). Previous studies showed that PD-1 expression on virus-specific T cells is the primary marker of exhaustion (82). The expression of PD-1 on CD8^+^ T cells is significantly higher in HIV-INRs than IRs (93, 94), suggesting that INRs have more exhausted CD8^+^ T cells. Similarly, Li et al. found that the frequencies of PD-1^+^CD39^+^ CD8^+^ T cells are negatively correlated with CD4^+^ T cell counts and the CD4/CD8 ratio in ART naïve patients (125).

However, in contrast to T cell exhaustion, the concept of CD8^+^ T cell senescence induced by HIV remains controversial (118). Especially in INRs, the study found that CD8^+^ memory, T_EM_, and T_TE_ cells showed lower markers of senescence than those of the IRs (26). One possible reason is that HIV inhibited the process of terminal differentiation and proliferation of CD8^+^ T_EM_ cells, expanded the less-differentiated transitional memory and CD28^–^CD57^–^CD8^+^ T cells, therefore decreasing the proportion of CD28^–^CD8^+^ T cells that express CD57 (126).

### 2.5 Double-negative T cells

Double-negative (DN) T cells represent a small subpopulation of approximately 3-5% of T lymphocytes in peripheral blood (127, 128). They are CD3 positive, CD4 and CD8 negative, express either TCR alpha and beta (αβ) or TCR gamma and delta (γδ), but do not express NK T cell markers (129). Although lacking certain phenotypic classification, DN T cells can be divided into naïve and active cells according to the transcriptome landscape (130). It can also be classified according to different functions, such as DN Tregs that can secrete anti-inflammatory cytokines and exhibit remarkably potent immunosuppressive potential (129, 131), T helper (Th)-like DN T Cells that can secrete cytokines to exert Th function and exhibit either protective or pathologic functions in different infections (129, 132, 133), cytotoxic DN T cells that mediate the killing effect of malignant tumors (134, 135).

In SIV/HIV infection, studies have found that DN T cells are associated with disease progression (54, 133, 136). Milush et al. reported that DN T cells with Th-like helper functions may compensate for the very low levels of CD4^+^ T cells in SIV-infected sooty mangabeys that were free of clinical AIDS for a long time (133). However, Liang et al. demonstrated that the numbers of DN T cells in HIV-infected patients with CD4^+^ T cells <250 cells/μL were significantly lower than those with CD4^+^ T cells between 250-500 as well as >500 cells/μL (54). Another study found that HIV-INRs had a low level of DN T cells, and the number of these cells was positively correlated with CD4^+^ T cell counts but negatively correlated with immune activation (55). Moreover, the production of transforming growth factor (TGF)-β1 by DN T cells might participate in the downregulation of immune activation after long-term ART (55), suggesting that DN T cells may play a role in immune reconstitution by regulating the immune response. On the contrary, Wang et al. found that the frequency of DN T cells was comparable between INRs and IRs, and had no correlation with immune activation, while only CD73^+^ DN T frequency was positively correlated with CD4^+^ T cell counts (137). It is suggested that there may be other mechanisms in the participation of DN T cells in immune reconstitution.

**Figure 2:**
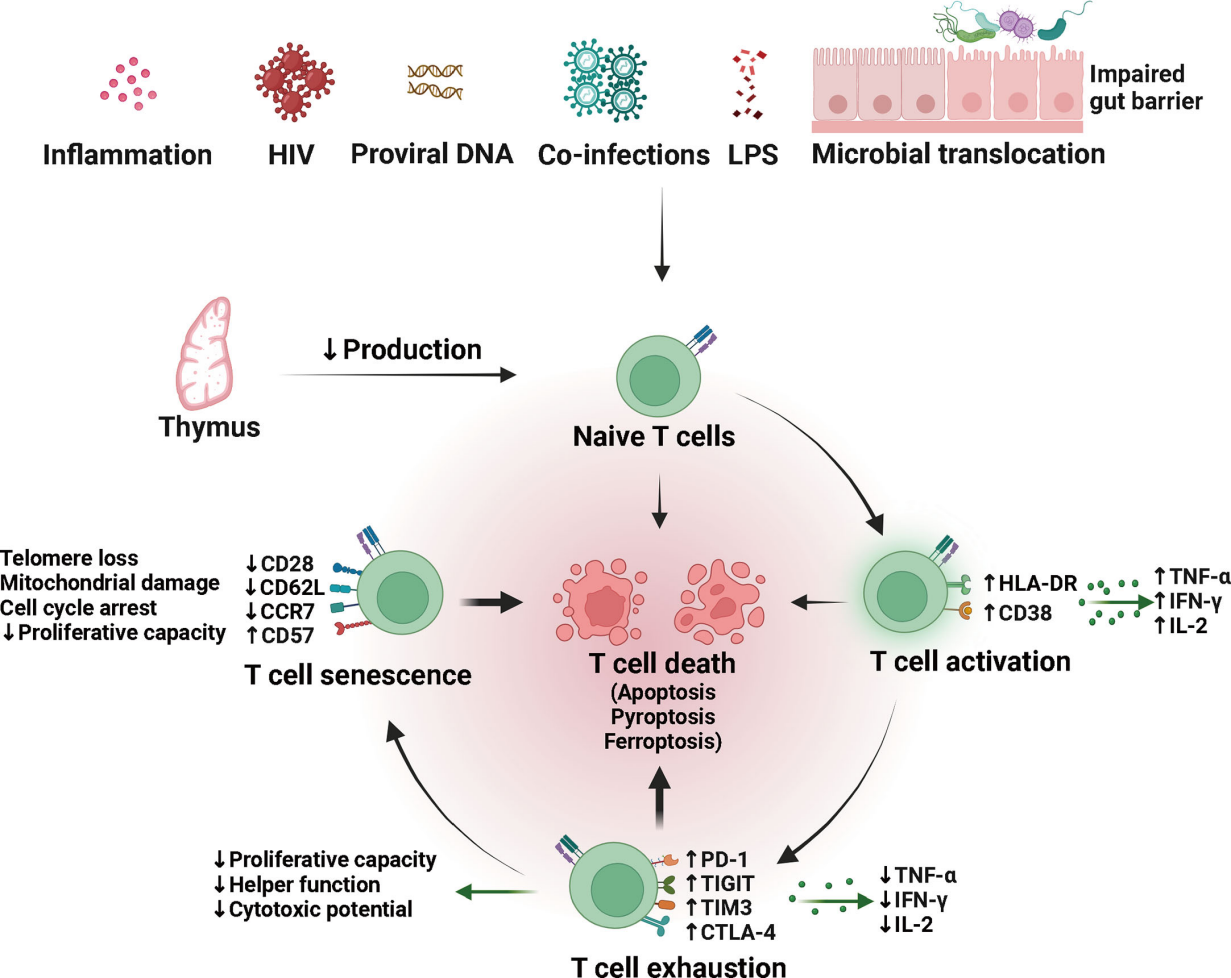
Quantity and quality alterations of T cells in HIV/AIDS patients with incomplete immune reconstitution. The naïve T (T_N_) cells produced by the thymus can be activated by multiple factors, including persistent viral reservoir, low residual viremia, co-infections, microbial translocation and dysbiosis. Difficult-to-remove risk factors may lead to persistent chronic immune activation, contributing to the development of T cell exhaustion and immune senescence. Exhausted and senescent T cells are qualitatively altered and prone to death, possibly through apoptosis, pyroptosis, and ferroptosis. LPS, lipopolysaccharide; HLA-DR, human leukocyte antigen DR; TNF-α, tumor necrosis factor-alpha; IFN-γ, interferon-gamma; IL-2, interleukin-2; PD-1, programmed death-1; TIGIT, T cell immunoglobulin and ITIM domain; TIM3, T cell immunoglobulin domain and mucin domain-containing protein 3; CTLA-4, cytotoxic T lymphocyte antigen-4.

To sum up, CD4^+^ T cells were decreased in quantity and altered in quality in HIV- INRs receiving antiviral therapy (**Figure 2**). Among them, the decrease in production and the increase in destruction of CD4^+^ T cells are the direct reasons for incomplete immune reconstitution. The abnormal quality is characterized by T cells’ overactivation, exhaustion, senescence, and susceptibility to death, resulting in weakened cell proliferation and differentiation, increased secretion of inflammatory molecules, and decreased secretion of anti-inflammatory cytokines. Furthermore, suboptimal CD4^+^ T recovery is associated with impaired homeostasis of other T cells such as CD8^+^ T cells, Tregs, and DN T cells. Although the abnormal quality of CD4^+^ T cells and impaired homeostasis of other T cells are not the direct cause of incomplete immune reconstitution, they may be involved in its pathological process. And the specific mechanism needs to be further studied.

## 3 Other immune cells

### 3.1 Natural killer cells

Human NK cells are broadly distributed innate lymphocytes (138, 139), including three common subpopulation groups: CD56^bright^CD16^dim/−^ subpopulation which is typically viewed as immature precursors and primarily secretes cytokines, the larger CD56^dim^CD16^bright^ subpopulation which is considered as mature subset with toxic effects, and the dysfunctional CD56^–^CD16^bright^ subpopulation with low cytotoxic activity and cytokine production (138, 140). In HIV infection, NK cells play a negative regulation as well as protective function (141), and may be associated with immune reconstitution. Bayigga et al. found that INRs had a higher proportion of pro-inflammatory CD56^bright^CD16^dim/−^ NK cells than IRs, while the largest CD56^dim^ NK cell subset was comparable among INRs and IRs (142). Similarly, another study showed that INRs exhibit an accumulation of autoreactive CD56^bright^ NK cells, possibly linked to decreased homeostatic control by Tregs, which contributes to incomplete immune reconstitution (143). However, Luo et al. reported that the absolute number, percentage, and subpopulation percentage of NK cells were similar between INRs and IRs, while the increased CD56^dim^CD16^+^ NK cell activation was predominantly in INRs and inversely correlated with the peripheral CD4^+^ T cell counts (144). Recently, the existence of a population of CD56^dim^CD16^dim/−^ NK cells was detected and found to be significantly higher in INRs than IRs (145). In addition to increased proportion, the killing ability of CD56^dim^ NK cells was also significantly increased in INRs, and significantly correlated with apoptosis of T lymphocytes (146). Although the above studies are not identical, they consistently indicate that NK cells play a negative role in immune reconstitution.

### 3.2 B cells

B cells are generated from stem cells in the bone marrow, and enter the periphery at an immature/transitional stage after forming a fully functional B-cell receptor, then develop into naïve B cells after further selection, accompanied by increased expression of CD21 (147, 148). Once a naïve B cell migrates into peripheral lymphoid tissues and encounters an antigen, its response can be divided into two ways: one that occurs without T-cell help, and one that occurs with T-cell help typically within the microenvironment of the germinal center (147). Affinity-matured B cells that exit the germinal center either serve as memory B cells or as long-lived plasma cells (148). In HIV infection, B cell-mediated immune response is sustained by HIV-specific memory B cells and plasma cells (148). A recent study found that the proportions of naïve B, memory B, and plasma cells are not associated with immune recovery, while the low frequency of CD21^+^ memory B cells is a risk factor for incomplete immune reconstitution (149). This may be related to the dysregulation of memory B cells and circulating T follicular helper cells (149, 150). In addition, another study found that the diversity of the B-cell receptor repertoire in HIV-INRs was decreased, and naïve B cells with low differentiation improve the immune reconstitution (151). These findings underscore the critical role of B cells in immune reconstitution after HIV infection.

### 3.3 Monocytes/macrophages and dendritic cells

Monocytes/macrophages act as first responders in innate immunity and then as mediators for adaptive immunity to help clear infections (152). In performing these functions, the macrophage inflammatory responses may also contribute to the pathogenesis. Stiksrud et al. found that HIV-infected individuals with suboptimal immune recovery exhibited more activated monocytes and dendritic cells (DCs) compared to individuals with adequate immune recovery (153). The persistent inflammation and activation of monocytes and other innate immune cells are likely associated with the persistent T cell activation and impaired effector functions in adults receiving antiretroviral therapy (154). Thus, it is suggested that monocytes/macrophages and DCs may be involved in the pathological process of changes in the quality of CD4^+^ T cells.

## 4 Soluble mediators and cytokines

### 4.1 Mechanisms of soluble biomarkers production

**Figure 3:**
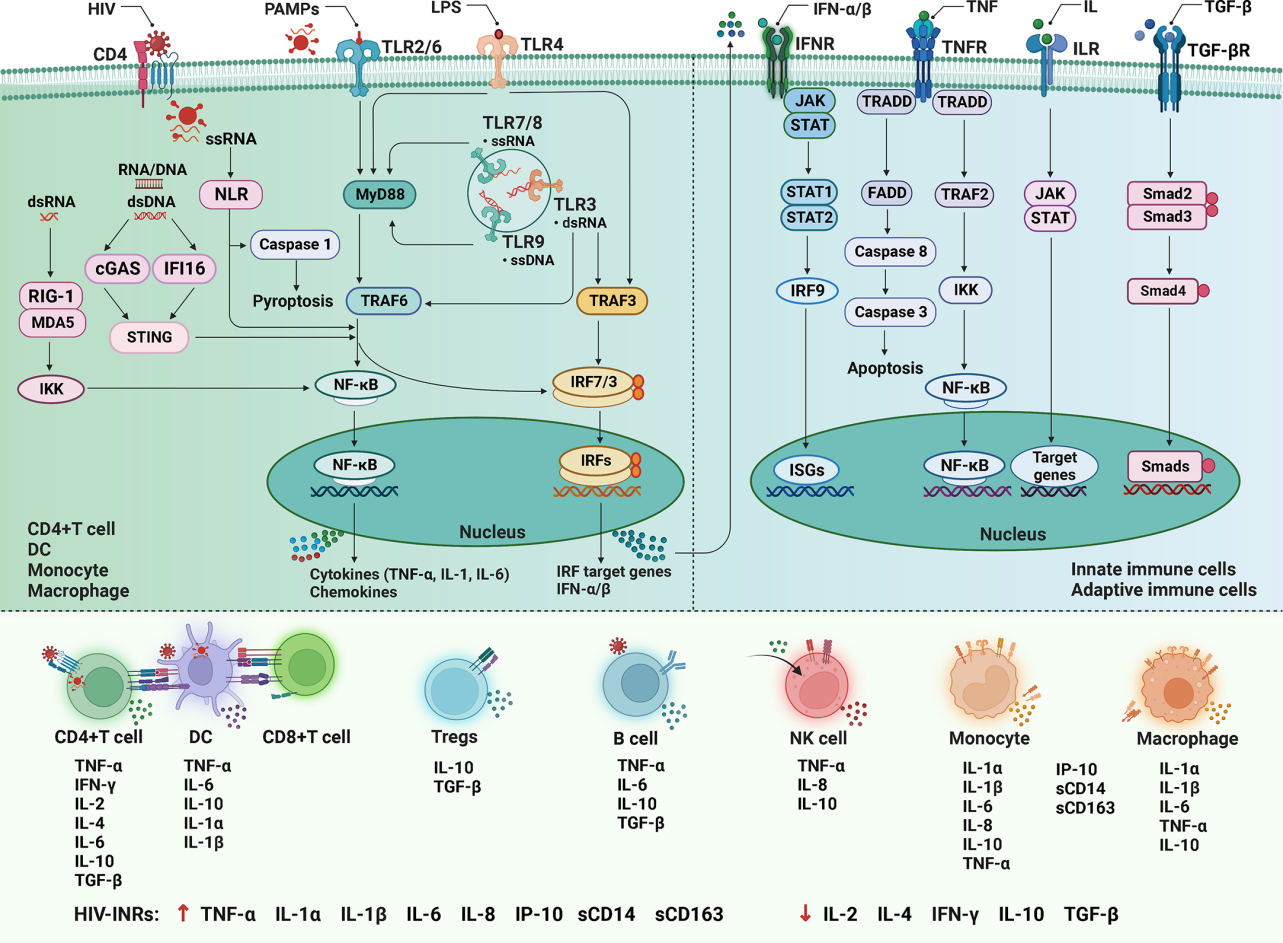
The cytokine signaling in HIV infection. After HIV infection, the pathogen-associated molecular patterns (PAMPs) are sensed by the pathogen-recognition receptors (PRRs) to trigger the innate immune response. Among them, the Toll-like receptors (TLRs) signal transduction pathways mainly include myeloid differentiation primary response gene 88 (MyD88) dependent and independent pathways. In the former, TLRs combine with MyD88 to recruit tumor necrosis factor (TNF) receptor-associated factor (TRAF)-6, and activate nuclear factor (NF)-κB and IFN regulatory factor (IRF)-7, induce the expression of pro-inflammatory factors and type I interferons (IFN-I); in the latter, TLRs sense HIV products and recruit TRAF3 and TRAF6, then activate NF-κB and IRF3. The nucleotide-binding oligomerization domain-like receptors (NLRs) can sense single-stranded RNA (ssRNA), and can also interact with caspase-1 to induce pyroptosis. The RIG-I-like receptors (RLRs) can sense the double-stranded RNA (dsRNA) to recruit I κB kinase (IKK) and activate NF-κB and IRF-3. The cyclic GMP/AMP synthase (cGAS) and IFN-inducible protein 16 (IFI16) can bind to double-stranded DNA (dsDNA), then activate the stimulator of interferon genes (STING) signal pathways. Initiation of the innate immune response produces a variety of soluble factors that recruit and activate innate immune cells. In addition, IFN-I, TNF, interleukin (ILs), and transforming growth factor (TGF)-β respectively bind to cell surface receptors and activate downstream signaling pathways to participate in immune regulation. LPS, lipopolysaccharide; MAD5, melanoma differentiation-associated protein 5; JAK-STAT, Janus kinase and signal transducer and activator of transcription proteins; ISGs, interferon-stimulated genes; TRADD, TNF receptor 1 (TNFR1)-associated death domain protein; FADD, Fas-associated protein with death domain; DC, dendritic cell; NK, natural killer cell; INRs, immunological non-responders.

The immune response to HIV infection begins with infected CD4^+^ innate immunocytes and CD4^+^ T cells, where the pathogen-associated molecular patterns (PAMPs) in viral products are sensed by the pathogen-recognition receptors (PRRs) of the host cell to trigger the innate immune response (155). The PRRs include the Toll-like receptors (TLRs), NLRs, RIG-I-like receptors (RLRs), and the novel DNA sensor cyclic GMP/AMP synthase (cGAS) as well as IFN-inducible protein 16 (IFI16) (156–158). Among them, TLR is located on the surface of various immunocytes and intracellular organelle membranes. Its signal transduction pathways mainly include myeloid differentiation primary response gene 88 (MyD88)-dependent pathways and MyD88-independent pathways (**Figure 3**). TLR2/6 is dependent on MyD88, TLR3 is independent of MyD8, and TLR4 employs both signaling pathways (159). In MyD88-dependent pathways, TLRs combine with intracellular junction protein MyD88, then recruit the downstream TNF receptor-associated factor (TRAF)-6, and activate nuclear factor (NF)-κB and IFN regulatory factor (IRF)-7, induce the expression of pro-inflammatory factors and type I interferons (IFN-I) (159, 160). In MyD88- independent pathways, TLRs sense HIV products and recruit downstream TRAF3 and TRAF6, then activate NF-κB and IRF3 to trigger the production of inflammatory factors (161). NLRs represent a diverse family of PRRs expressed in the cytosol of various cell types, which can sense viral single-stranded RNA and signal to the activation of NF-κB and IRF3, and can also interact with caspase-1 upon activation to induce pyroptosis (162, 163). RLRs are another important class of PRRs that can sense the double-stranded RNA, then binds Cardif in the mitochondria to recruit I κB kinase (IKK), and activate NF-κB and IRF-3 to trigger IFN-I production to exert antiviral effect (159, 164). IFI16 and cGAS can sense and bind to cytosolic double-stranded DNA, then activate the stimulator of interferon genes (STING) signal pathways (157, 158).

Initiation of the innate immune response, in addition to antiviral defense, produces a variety of soluble factors, including IFN-I, inflammatory cytokines and chemokines. These soluble factors recruit and activate innate immune cells, including DCs, monocytes/macrophages and NK cells (155). In addition, IFN-I, TNF, ILs, and TGF-β respectively bind to cell surface receptors, activate downstream signaling pathways, and play several essential roles, such as inducing and regulating the development, differentiation, survival, and function of myeloid and lymphoid cells, activating and regulating adaptive immune response, and participating in inflammatory response (165).

### 4.2 T cell-related cytokines

Chronic infection and systemic inflammation lead to persistent activation of the immune system, and activated lymphocytes are consumed, which affects the secretion of cytokines. As reported in previous studies, HIV-INRs had a low level of certain cytokines than that in IRs, including IL-2, IL-4, IL-10, and IFN-γ (143, 146, 166). These cytokines have been proven to be closely correlated to the activation and proliferation of T lymphocytes (167–169). Thus, the decrease of cytokine production by lymphocytes in INRs eventually leads to T cell depletion (146). In theory, improving the regulation of T cell-related cytokines may be beneficial for INRs. For example, treatment with IL-2 in HIV-infected patients can significantly increase CD4^+^ T cell counts and enhance immune function (170).

IL-7 is also crucial in T cell homeostasis as it maintains T cell survival, induces proliferation, and promotes *de novo* production (171, 172). The IL-7 responsiveness is largely dependent on the presence or absence of the IL-7 receptor (IL-7R). But unlike the cytokines mentioned above, IL-7 is mainly produced by bone marrow and thymic stromal cells (173). In HIV-infected patients, the level of IL-7 is higher and the level of IL-7R is lower than that in healthy controls (174, 175), and there is a negative correlation between plasma IL-7 levels and CD4^+^ T cell counts (176). Consequently, INRs exhibit a higher stromal production of IL-7, a diminished expression of IL-7R and a reduced IL-7 mediated proliferation responsiveness compared to normal responders (177–180). Moreover, the reduction of naïve and thymic naïve CD4^+^ T cells in INRs is associated with increased serum IL-7 levels and decreased IL-7R expression (24). Down-regulation of IL-7R is related to T cell activation and is a main factor influencing the restoration of CD4^+^ T cells (181). Marziali et al. also found that the reduced expression of IL-7Rα was associated with persistent immune activation and the alteration of Treg frequencies, which in part explains the low level of CD4^+^ T cells observed in INRs (24). Another study found that the IL7RA polymorphisms seem to predict the CD4^+^ T cell recovery in HIV-infected patients on ART (182). These studies indicate that dysregulation of IL7/IL7R homeostasis plays an important role in incomplete immune reconstitution. Thus, IL-7-based therapy, combined with efficient ART, may be beneficial to HIV-INRs by promoting thymic output, inducing a sustained increase of T_N_ and T_CM_ cell counts, thereby enhancing T cell recovery (183–185).

### 4.3 Innate immunity activation markers

Soluble CD14 (sCD14) is part of innate immunity and plays an important role in the inflammatory response induced by lipopolysaccharide, an outer cell wall component of gram-negative bacteria. Plasma sCD14 forms a complex with lipopolysaccharide, then binds to LPS receptors on monocytes/macrophages, and activates the cells to produce pro-inflammatory cytokines (186). A series of studies have shown that HIV-infected patients have higher levels of sCD14 than healthy subjects (187–189), and plasma sCD14 are independently associated with disease progression (188, 190). Dunham et al. found that plasma sCD14 levels from INRs were significantly higher than that in HIV-negative subjects, while the difference between INRs and IRs was not significant (191). Moreover, the concentrations of sCD14 during ART were inversely associated with subsequent CD4^+^ T cell counts (191, 192), and were also correlated with blood inflammatory markers, shorter telomeres, and increased Treg levels (98, 191).

Soluble CD163 (sCD163) is another hallmark of monocyte/macrophage activation. It was higher in the plasma of chronic HIV-infected patients than in healthy subjects, and decreased after effective ART but did not return to HIV-seronegative levels (193). The high level of plasma sCD163 was correlated with gut mucosal disruption, positively correlated with the percentage of CD14^+^CD16^+^ monocytes and T cell activation markers (193, 194), and increased sCD163 may serve as a marker of immunosenescence (195). Moreover, Fischer-Smith et al. found a strong inverse correlation between CD163^+^/CD16^+^ monocyte and the number of CD4^+^ T cells below 450 cells/μL (196). Plasma sCD163 levels were also inversely correlated with CD4^+^ T cell percentage and CD4/CD8 ratio (194).

Interferon-γ-induced protein 10 (IP-10 or CXCL-10) is a chemokine involved in trafficking immune cells to inflammatory sites. It is produced by various cell types on stimulation, while monocytes are responsible for the greatest proportion of IP-10 expression (197). Plasma IP-10 levels are significantly higher in HIV-infected patients than that in healthy subjects (198–201), and inversely related to CD4^+^ T cell counts, regardless of those pre- or post-ART (200, 202). IP-10 levels are also associated with the time for CD4^+^ T cell counts to fall below 200 cells/μL during Fiebig stages III-V (203). In addition, elevated IP-10 levels are associated with immune activation and can promote the progression of inflammation (197, 204). Meanwhile, exposure to persistent IP-10 leads to a decrease in the number of CD4^+^ and CD8^+^ T cells capable of producing cytokines, a decrease in T cell proliferation, and can effectively impair NK cell function (198, 205).

Taken together, these studies suggest that monocyte/macrophage activation, marked by increased expression of sCD14, sCD163, and IP-10, is associated with T cell activation, immune senescence, and impaired immune cell function in HIV-infected patients, which is involved in the occurrence of incomplete immune reconstitution and may be an intervention measure for INRs.

### 4.4 Key pro-inflammatory cytokines

Immune activation and systemic inflammation are usually accompanied by the secretion of soluble inflammatory mediators, such as pro-inflammatory cytokine TNF-α, IL-1β, IL-6, and IL-8. Among them, plasma IL-6 levels were found to be elevated in HIV-INRs before ART as well as after virological suppression (206, 207), and were negatively correlated with CD4^+^ T cell counts (208). In addition, the intestinal microbiota of HIV/AIDS patients was disordered, and the number of intestinal flora was correlated with the number of CD4^+^ T cells and the levels of TNF-α and IL-6 (209). Shive et al. found that IL-6 can induce low-level cycling of T_N_ cells, IL-1β can induce cell cycling and turnover of memory CD4^+^ T cells, and both cytokines can decrease T cell surface expression and RNA levels of IL-7 receptor (57). Moreover, the induction of CD4^+^ T cell turnover and diminished T cell responsiveness to IL-7 by IL-1β and IL-6 exposure may contribute to the lack of CD4^+^ T cell reconstitution in HIV-infected subjects on ART (57). These studies demonstrate that pro-inflammatory cytokines negatively affect the quantity of CD4^+^ T cells.

### 4.5 Anti-inflammatory cytokines

In addition to IL-10, TGF-β is another major anti-inflammatory cytokine that controls the development, differentiation, and function of Tregs (210). Younes et al. showed that the expression of genes for the TGF-β signaling pathway (TGIF1, SMAD1, SMAD7, LEFTY) was lower in HIV-INRs (58), and the production of TGF-β by Tregs was impaired in the setting of incomplete immune reconstitution (58). Another study also found that plasma TGF-β levels were significantly lower in the INRs when compared to plasma levels in the IRs (211). In addition, plasma levels of TGF-β were negatively correlated with T cell exhaustion and senescence phenotypes, and positively correlated with CD4^+^ T cell counts in INRs (211). These studies indicate that low levels of anti-inflammatory cytokines are associated with impaired function of Tregs and difficulty in controlling inflammation, which may participate in the occurrence of incomplete immune reconstitution in HIV-infected patients.

### 4.6 Other biomarkers associated with immune reconstitution

HIV infection induces widespread expression of IFN-I and IFN-stimulated genes (212, 213), and the abnormally elevated IFN-I signaling persists in some patients even under extensive ART (191, 214). Chronic exposure to IFN-I hampers the reversion of hyperimmune activation and immune recovery in INRs (191, 213). Therefore, targeting IFN-I mediated activation may provide a potential strategy to enhance T cell recovery (215, 216).

C-reactive protein (CRP) is a prophylactic acute-phase plasma protein and a non-specific marker of systemic inflammation that is stimulated by cytokines such as IL-6, IL-1, and TNF to be produced in the liver. The levels of CRP were significantly higher in INRs than that in IRs (217), and were inversely associated with CD4^+^ T cell counts (192). For hypersensitive CRP (hsCRP), there was no difference between INRs and IRs (26). However, the INRs showed significantly higher levels of hsCRP in comparison with healthy subjects (218), and INRs showed a tendency to have more subjects with hsCRP levels exceeding 2 μg/mL or 3 mg/dL when compared with IRs (26, 218).

Additionally, soluble TNF receptors, sTNF-RI, and sTNF-RII, were measured in plasma as biomarkers of TNF activity. Dunham et al. found that although the difference in sTNF-RI and sTNF-RII levels between INRs and IRs did not reach statistical significance, the levels of sTNF-RII in INRs were higher than those found in healthy subjects and were more comparable to those observed in viremic subjects (191).

## 5 Conclusions and perspectives

In summary, CD4^+^ T cell homeostasis alteration in HIV-infected subjects with incomplete immune reconstitution despite successful viral suppression during ART, including decreased quantity and altered quality. Additionally, suboptimal CD4^+^ T cell recovery is associated with impaired homeostasis of multiple immunocytes such as CD8^+^ T cells, Tregs, DN T cells, NK cells, B cells, monocytes/macrophages and DCs, as well as abnormal secretion of various soluble mediators and cytokines. While these data are impressive and informative, there is limited understanding of the primary causes of incomplete immune reconstitution and the causal relationship of immunocytes or soluble mediators to incomplete immune reconstitution. This will be a meaningful research direction that can help to identify INRs earlier and provide physicians with optimal strategies to improve CD4^+^ T cell recovery. When solving the above problems, it should be noted that different individuals may have different primary causes of incomplete immune reconstitution. Methodologically, immunology, genomics, transcriptomics, and single-cell sequencing methods are useful tools in this regard. Moreover, future more in-depth mechanistic and clinical studies are needed to develop immune-based interventions for incomplete immune reconstitution.

## Author contributions

LY, FZ and XY were involved in the conception of the study. LY wrote the draft of the review. KX, QX, LT, TL, SW and RY revised the manuscript. All authors contributed to the article and approved the submitted version.
